# Dynamic Failure Experimental Study of a Gravity Dam Model on a Shaking Table and Analysis of Its Structural Dynamic Characteristics

**DOI:** 10.3390/s24051602

**Published:** 2024-02-29

**Authors:** Jianchun Qiu, Wenqin He, Dongjian Zheng, Yanxin Xu, Shaolong Guo, Tianxiao Ma, Pengcheng Xu, Yongtao Liu

**Affiliations:** 1 College of Hydraulic Science and Engineering, Yangzhou University, Yangzhou 225009, China; mz120231134@stu.yzu.edu.cn (W.H.); m18994113495@163.com (P.X.); 2 State Key Laboratory of Hydrology-Water Resources and Hydraulic Engineering, Hohai University, Nanjing 210098, China; zhengdj@hhu.edu.cn (D.Z.); hhu.xyx@163.com (Y.X.); dream2020@tcu.edu.cn (S.G.); liuyongtao_hhu@outlook.com (Y.L.); 3 National Engineering Research Center of Water Resources Efficient Utilization and Engineering Safety, Hohai University, Nanjing 210098, China; 4 College of Water-Conservancy and Hydropower, Hohai University, Nanjing 210098, China; 5 College of Civil Engineering, Tianjin Chengjian University, Tianjin 300384, China; 6 College of Water Conservancy, Shenyang Agricultural University, Shenyang 110161, China; matianxiao@syau.edu.cn

**Keywords:** gravity dam, dynamic failure experiment, shaking table, dynamic response, dynamic characteristic parameters, damage

## Abstract

Investigating the dynamic response patterns and failure modes of concrete gravity dams subjected to strong earthquakes is a pivotal area of research for addressing seismic safety concerns associated with gravity dam structures. Dynamic shaking table testing has proven to be a robust methodology for exploring the dynamic characteristics and failure modes of gravity dams. This paper details the dynamic test conducted on a gravity dam model on a shaking table. The emulation concrete material, featuring high density, low dynamic elastic modulus, and appropriate strength, was meticulously designed and fabricated. Integrating the shaking table conditions with the model material, a comprehensive gravity dam shaking table model test was devised to capture the dynamic response of the model under various dynamic loads. Multiple operational conditions were carefully selected for in-depth analysis. Leveraging the dynamic strain responses, the progression of damage in the gravity dam model under these diverse conditions was thoroughly examined. Subsequently, the recorded acceleration responses were utilized for identifying dynamic characteristic parameters, including the acceleration amplification factor in the time domain, acceleration response spectrum characteristics in the frequency domain, and modal parameters reflecting the inherent characteristics of the structure. To gain a comprehensive understanding, a comparative analysis was performed by aligning the observed damage development with the identified dynamic characteristic parameters, and the sensitivity of these identified parameters to different levels of damage was discussed. The findings of this study not only offer valuable insights for conducting and scrutinizing shaking table experiments on gravity dams but also serve as crucial supporting material for identifying structural dynamic characteristic parameters and validating damage diagnosis methods for gravity dam structures.

## 1. Introduction

Concrete gravity dams, as common river impoundment hydraulic structures, are widely employed due to their high stability, reliability, and adaptability to complex terrains [1,2,3]. However, during prolonged operation and management, concrete gravity dams may experience varying degrees of structural damage or pathological issues [4,5]. In seismic zones, concrete gravity dams are particularly susceptible to accumulating structural damage due to frequent earthquakes [6]. Seismic loads can lead to gradual structural damage, weakening a dam’s stiffness and integrity, thus reducing its ability to withstand external loads and posing a threat to dam safety [7]. In seismic high-intensity areas, severe concrete dam accidents not only result in significant damage to the surrounding facilities but also pose a substantial threat to the life and property of downstream residents [8,9]. Therefore, it is necessary to study the dynamic response patterns and failure mode patterns of concrete gravity dams subjected to strong earthquakes to evaluate their seismic safety [10,11].

The present methods for investigating the dynamic response and damage pattern of concrete dams during seismic loading primarily involve prototype dam observation [12], shaking table testing, and numerical simulation. In recent decades, researchers have invested substantial efforts in numerically simulating the dynamic response and failure modes of gravity dams under seismic loads. For example, Asterus PG and Tzamtzis AD [13] developed a numerical simulation method for the dynamic response analysis of concrete gravity dam–reservoir systems, and the simulation results demonstrated that the dynamic response of a gravity dam was substantially influenced by the interactions at the interfaces between the contacting materials. Qiu Jianchun et al. [14] conducted a numerical simulation of a concrete arch dam under seismic loads; the concrete damaged plasticity model was adopted as the constitutive model for the dam body, and the dynamic responses and damage pattern of the arch dam were obtained. They also conducted the shaking table testing of an arch dam model, and crack damage appeared and developed in the middle of the arch crown. Based on the measured dynamic responses, a recursive TVARX approach was proposed to identify the damage of the two arch dam examples. Due to the influence of various complex factors on gravity dams, such as their structural form, load conditions, boundary conditions, foundation rock conditions, and environmental conditions, numerical analyses often need to be built on specific assumptions [15]. However, variations in nonlinear analysis constitutive models and parameter values lead to diverse numerical analysis outcomes for the dynamic response of dams under seismic conditions. Additionally, there are limited instances of high concrete gravity dams collapsing or failing directly due to seismic effects, and research on the failure mechanisms and damage patterns of high dams is not still comprehensive. Shaking table testing is an effective method for studying the nonlinear dynamic response and damage mechanisms of structures, providing validation and a meaningful supplement to theoretical analysis and numerical simulation calculations [16,17]. Conducting such tests is crucial for comprehensively understanding a dam’s dynamic response and failure modes under strong seismic action, providing the validation of the numerical analysis results, and offering effective support for the seismic design of dams.

To explore the dynamic characteristics and failure patterns of gravity dams subjected to intense seismic loads, scholars have undertaken experimental investigations utilizing shaking table models of gravity dams. For example, Chen Jianyun et al. [18] conducted a dynamic model test on a shaking table for a concrete gravity dam, comprehensively examining the entire process of the dam, including elastic deformation, damage, and failure under different levels of peak ground acceleration. Xu Qiang et al. [19] conducted shaking table tests to investigate the seismic failure of the reinforced and unreinforced monoliths of the Huangdeng gravity dam, using emulation concrete material and fine alloy wire to simulate dam concrete and steel reinforcement in experiments. Mridha Subrata et al. [20] conducted a shaking table model experiment on the Koyna concrete gravity dam, employing a 1:150 scale ratio on a horizontal shaking table with sinusoidal wave motion. For the experiment, a model material comprising cement, sand, bentonite, and water was blended to adhere to the laws of similitude. The dynamic response, crack formation, crack opening, sliding along crack planes, and stability after crack formation of the dam model were studied in the study. Wang et al. [21] chose a 203 m high gravity dam as an example and considered the interaction between the dam body and reservoir water; they compared the structural dynamic characteristics of gravity dams under seismic loading via shaking table model experiments and numerical simulations. On the other hand, some studies have focused on identifying dynamic parameters and damage in concrete dam structures. For example, Lin Cheng et al. [22] proposed an online modal parameter identification method for concrete dams using the subspace tracking-based technique, and a newly developed recursive stochastic subspace identification method based on the generalized subspace tracker algorithm was used to obtain the time-varying modal parameters of concrete dams during earthquakes. Zar Ali et al. [23] developed a damage identification method for concrete arch dams based on vibration analysis, employing least-square support vector machines and salp swarm algorithms, and a numerical arch dam model example was used to verify the effectiveness of the proposed method. These studies have enriched the structural health monitoring study of concrete dams under seismic loading.

Recognizing the pivotal role that shaking table testing plays in examining the seismic performance and failure patterns of structures [24], a proposed concrete gravity dam intended for construction in a high seismic zone was the focus of this study, with a planned height of 100 m. Through shaking table testing, the structural dynamic response and seismic vulnerability of the gravity dam were investigated. This approach not only offers essential seismic support for gravity dams but also serves as valuable validation material for damage identification algorithms in gravity dam structures. To achieve these objectives, this study leveraged insights from previous shaking table tests on gravity dams and referred to past model materials. An emulation concrete material cast with a combination of heavy crystal sand, heavy crystal powder, cement, and water was carefully designed and produced. The process of designing and fabricating the shaking tables for gravity dams, including the installation of accelerometers and strain gauges, was detailed in this study. The dynamic responses of the gravity dam model under several operational conditions were selected for analysis, and the examination was initiated by analyzing the damage characteristics and development of the gravity dam model based on the measured dynamic strain responses. Next, an analysis of the acceleration amplification coefficients and acceleration response spectrum characteristics was conducted based on the measured acceleration responses. Then, the modal parameters of the dam model under different conditions were identified by using numerical algorithms for subspace state space system identification (N4SID) [25], facilitating the analysis of the dynamic characteristics of the gravity dam models under various conditions. Moreover, the identified dynamic characteristic parameters were compared with the actual damage distribution and development of the gravity dam model, and the sensitivity of each recognized dynamic characteristic parameter to structural damage was also compared. This comparison provides valuable references for the shaking table model testing and dynamic characteristic parameter analysis of gravity dam structures. The results of this study not only provide valuable insights for executing and scrutinizing gravity dam shaking table experiments but also serve as essential substantiating material for the identification of structural dynamic characteristic parameters and the validation of damage diagnosis methods for gravity dam structures.

The remainder of this paper is organized as follows: Section 2 describes the design of the gravity dam experimental model on a shaking table. In Section 3, damage identification methods, including frequency spectrum characteristic parameters and the N4SID method, are described. In Section 4, the dynamic strain responses and acceleration responses were analyzed to identify the damage by using different dynamic characteristic parameters, and the sensitivities of these parameters to different levels of damage were compared and analyzed. Finally, Section 5 presents the conclusions drawn from this study.

## 2. Design of the Gravity Dam Experimental Model on the Shaking Table

### 2.1. The Emulation Concrete Material

**Figure 1:**
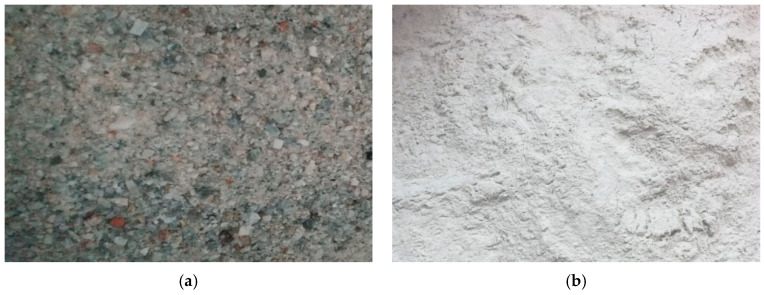
Heavy crystal sand and heavy crystal powder. (**a**) Heavy crystal sand. (**b**) heavy crystal powder.

Due to the geometric similarity scale of the gravity dam model being in the range of one-tenth to one-hundredth, the model material needs to possess characteristics such as a high density, a low dynamic elastic modulus, and appropriate strength. A model material, composed of heavy crystal sand, heavy crystal powder, cement, quick-setting agent, and water, was invented through pouring and mixing, and was named emulation concrete. The primary component of the heavy crystal sand and heavy crystal powder was barium sulfate, as shown in Figure 1, and the two kinds of material had a densities ranging from 4100 to 4200 kg/m^3^. The mass ratios of heavy crystal sand, heavy crystal powder, cement, water, and quick-setting agent during casting were 66.25%, 25.0%, 0.5%, and 0.05%, respectively. The average density of the specimens after the final casting and drying process was 3175 kg/m^3^.

**Figure 2:**
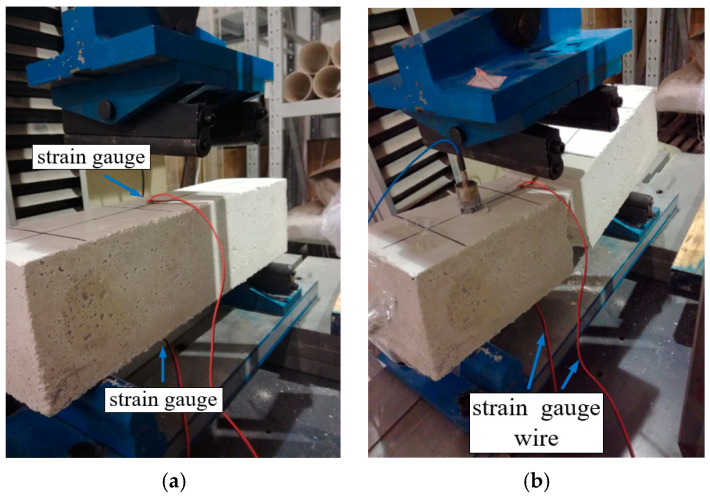
Flexural tensile strength measurements of the emulated concrete. (**a**) Before loading. (**b**) After loading.

To comprehend the mechanical properties of the emulation material, tests were conducted to determine the tensile strength and dynamic elasticity modulus of the emulation concrete materials. Beam specimens with dimensions of 100 mm × 100 mm × 400 mm were manufactured, and the tensile strength of the specified concrete samples was determined through a four-point bending test, as illustrated in Figure 2. The resulting average tensile strength of the emulation material was determined to be 68.7 KPa.

**Figure 3:**
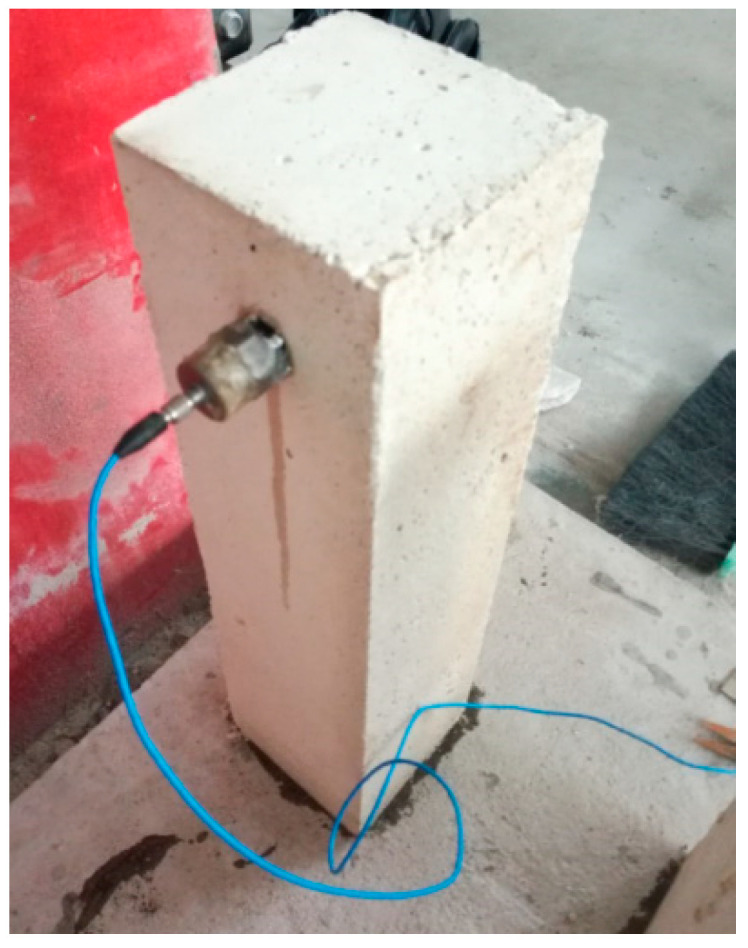
Dynamic elastic modulus measurement of the emulation concrete.

To obtain the dynamic elastic modulus of the emulation concrete, beam specimens measuring 100 × 100 × 400 mm were securely attached to the concrete bases with an epoxy resin adhesive. After a 24 h curing period, an accelerometer was affixed to the upper side of the beam specimens by 502 glue, as illustrated in Figure 3. Subsequently, the cantilever beam was laterally struck, and the resulting vibrational responses were recorded using the DSPACE devive (model RTX1003). Through the analysis of the collected vibration responses, the fundamental frequency of the cantilever beam could be ascertained, and the dynamic elastic modulus of the emulation concrete was subsequently calculated via inverse finite element analysis. The material’s dynamic elastic modulus measured using this method was compared with the results obtained from the dynamic tester, which showed a high level of consistency upon testing. Multiple sets of specimens were utilized for testing and analysis, yielding an average dynamic elastic modulus of 1.05 GPa for the material.

### 2.2. Gravity Dam Model Design and Sensor Placement

**Figure 4:**
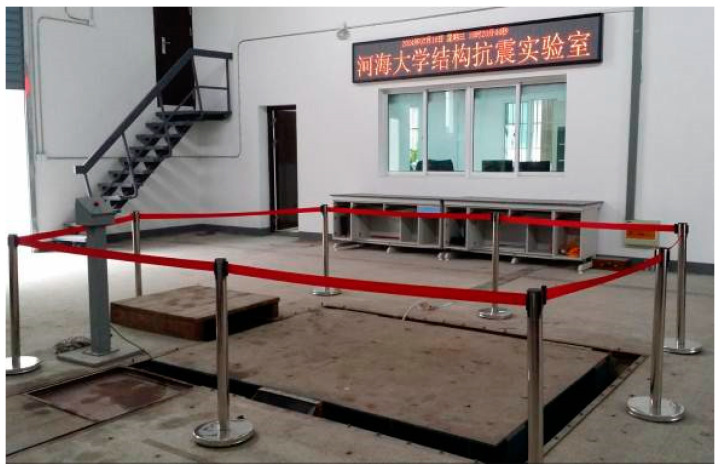
The shaking table.

**Figure 5:**
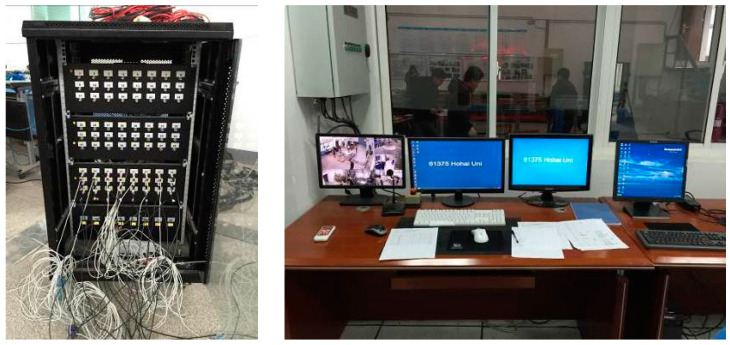
Data acquisition system.

The experiment was conducted in the Structural Seismic Laboratory at Hohai University. The excitation system used for the experiment was the simulated earthquake shaking table, as illustrated in Figure 4 and Figure 5. This system consisted of a hydraulic oil source, thrust actuator, shaking table body, cooling system, and digital control system. The table dimensions were 2.0 m by 2.8 m, with a maximum allowable load capacity of 6 tons. The operating frequency ranged from 0. 1 Hz to 100 Hz, with maximum horizontal and vertical accelerations of ±1.2 g and ±0.8 g, respectively. The maximum horizontal and vertical velocities were ±50 cm/s and ±35 cm/s, respectively.

Considering the experimental conditions and the mechanical properties of the developed emulation concrete material, a gravity dam model with a geometric ratio of 1/100 was designed for this study. The model features a dam height of 1.3 m, a width of 0.28 m, a downstream slope ratio of 1.25:1, and a downstream length of 0.12 m along the river. To simulate a partial foundation, the model incorporated a foundation thickness of 0.3 m, extending 0.3 m upstream and downstream. The overall model length along the river was 1.62 m, with a volume of 0.323 m^3^ and a mass of 1.01 tons.

**Figure 6:**
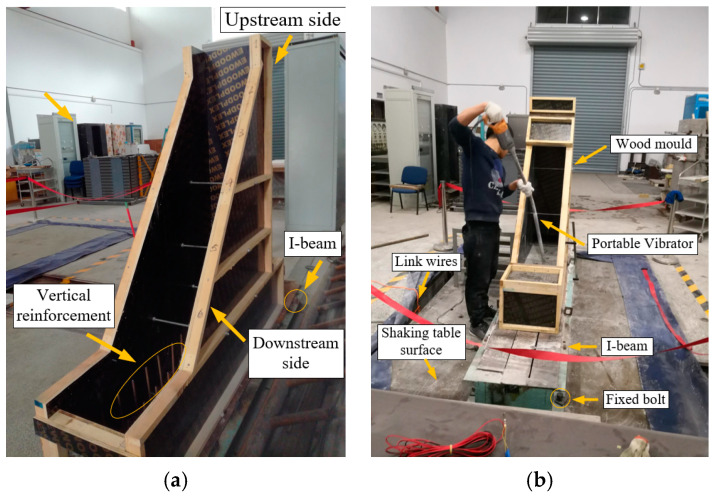
The wooden mold and gravity dam model casting. (**a**) The wooden mold. (**b**) Gravity dam model casting.

As illustrated in Figure 6, a wooden mold was used for casting the gravity dam model. The gravity dam model was poured onto an I-beam, and vertical steel bars with a length of 15 cm were welded onto the I-beams to enhance the connection between the I-beams and the model base, ensuring the smooth transmission of seismic excitation from the shaking table to the foundation of the gravity dam structure. The I-beam was securely fastened to the shaking table surface using bolts. The significantly greater stiffness of the I-beam enables the effective transmission of seismic excitation from the shaking table surface to the foundation of the gravity dam model, also serving to protect the shaking table surface.

The stiffness of the I-beam was significantly greater than that of the model material, allowing for the effective transmission of seismic excitation from the shaking table surface to the foundation of the gravity dam model. Additionally, the shaking table surface was protected. Before casting the model material, the entire interior of the wooden mold was first coated with a mold release agent to facilitate the subsequent smooth demolding, ensuring a smooth and clean surface for the model.

**Figure 7:**
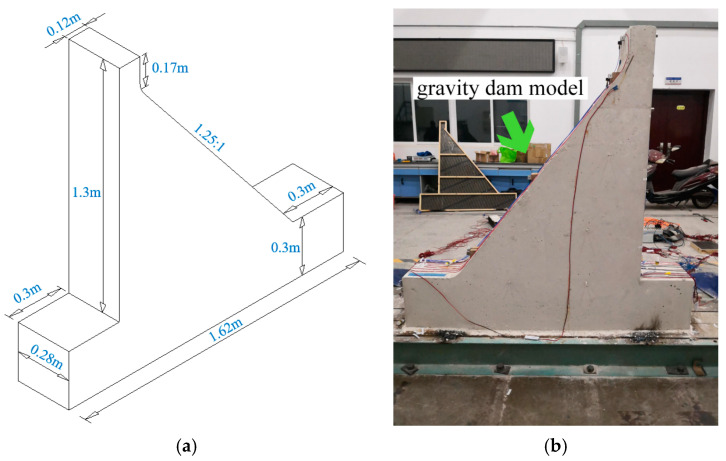
The gravity dam model. (**a**) Spatial diagram. (**b**) Gravity dam model casting.

Figure 7a shows the schematic dimensions of the entire gravity dam model, while Figure 7b shows the physical model of the complete gravity dam after demolding. Due to the constraints of the shaking table conditions, the influence of reservoir water was not considered.

**Figure 8:**
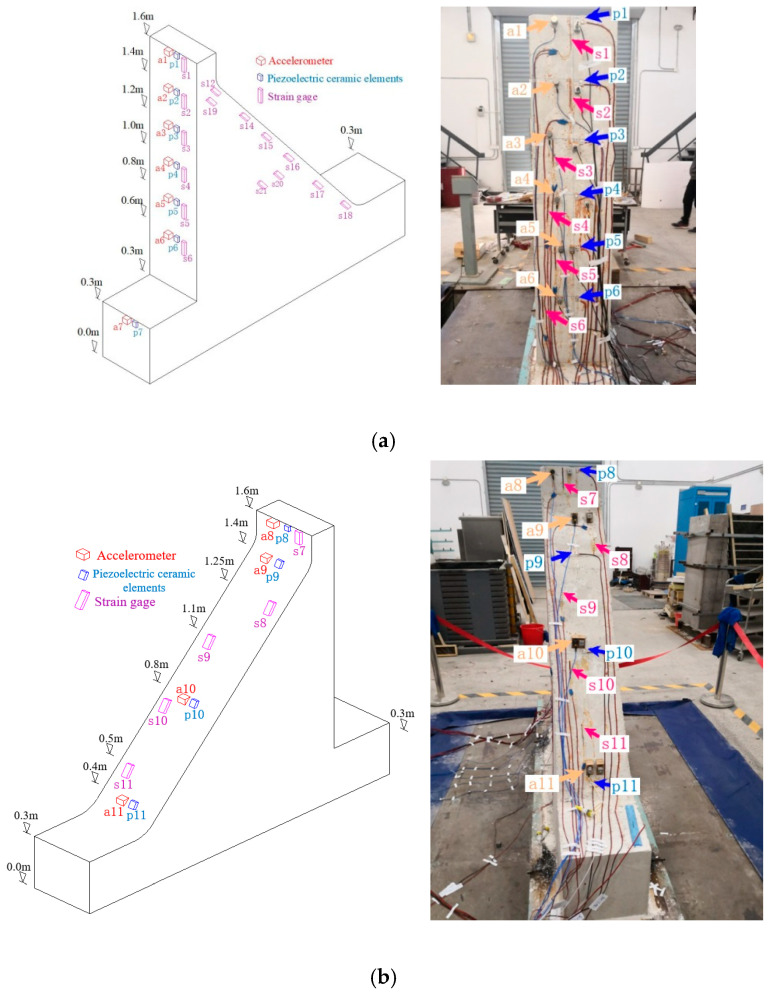
Schematic and physical diagrams of the sensor arrangement on the dam model. (**a**) Sensor arrangement on the upstream side of the gravity dam model. (**b**) Sensor arrangement on the downstream side of the gravity dam model.

The dynamic response of the gravity dam model was monitored by strategically placing accelerometers, piezoelectric ceramic elements, and strain gauges at different locations and elevations on the gravity dam model and connecting these sensors to the data acquisition system. Assuming a base elevation of 0.0 m, the dam base was positioned at an elevation of 0.3 m, while the dam crest was positioned at 1.6 m. To obtain the structural dynamic response of the gravity dam model at various locations under seismic loads, acceleration sensors and strain gauges were strategically placed at regular intervals on both the upstream and downstream sides of the gravity dam model. To distinguish the sensors placed at different locations, a unique identifier was assigned to each sensor. The layouts of the sensors, both schematic and physical, are illustrated in Figure 8. Additionally, an accelerometer marked as a0 was installed on the shaking table surface to monitor the acceleration response under various conditions. Before commencing the complete structural model experiment, tapping tests must be performed to validate the effectiveness and sensitivity of each sensor. This approach is essential for ensuring the accuracy of all connections and verifying that the wiring of each acquisition channel aligns with the correctly numbered sensor.

### 2.3. Experimental Procedure

Multiple dynamic loading conditions, including white noise conditions and artificial seismic wave conditions, were simulated in the experiment. The loading conditions for the model are listed in Table 1, with the seismic loads applied in the downstream direction. White noise conditions were used to determine the dynamic characteristics of the test model, while artificial seismic waves were used to assess the dynamic response of the arch dam model under different seismic wave intensities.

## 3. Damage Identification Methods

### 3.1. Frequency Spectrum Characteristic Parameters

The structural dynamic responses consist of various frequency bands. When damage occurs in a structure, its structural dynamic characteristics undergo changes, leading to variations in the spectral features of the structural dynamic response. To obtain the frequency-domain characteristics of structural dynamic response time histories, it was essential to convert dynamic response time histories from the time domain to the frequency domain. Fourier transformation facilitates spectral analysis, wherein the frequency components and their corresponding amplitudes within the responses can be scrutinized. This process elucidated the distribution of the signal in the frequency domain, offering crucial insights that were often more significant than those covered in the time domain. Specifically, this approach involves transforming the dynamic response signal, which evolves over time, into a spectrum that varies with frequency.

The forward transformation of the discrete Fourier transform is expressed by the following equation:

(1) $$Y(k)=\sum_{n=0}^{N-1}y(n)e^{-j2\pi nk/N}(n=0,1,2,\cdots ,N-1)$$

where N is the sampling point, k is the frequency, n is the index of the time-domain discrete values, and Y(k) is the Fourier-transform coefficient of the *k*-th harmonic.

To assess the variations in the spectral characteristics of the structural dynamic response, the following statistical parameters were defined based on the spectrum graph:

(2) $$R_{d}=\frac{\sum f_{i}A_{i}}{\sum A_{i}}/\frac{\sum f_{i0}A_{i0}}{\sum A_{i0}}$$

where $R_{d}$ is the proposed spectral statistical parameter indicator, $f_{i}$ represents the frequency of the i-th point in the dynamic response spectrum graph, $A_{i}$ represents the acceleration amplitude of the i-th point in the corresponding spectrum graph, $f_{i0}$ represents the frequency of the i-th point in the response spectrum graph of the measurement point arranged on the shaking table surface, and $A_{i0}$ is the acceleration amplitude of the i-th point in the response spectrum graph of the measurement point arranged on the shaking table surface.

### 3.2. Modal Parameter Identification Based on Numerical Algorithms for Subspace State Space System Identification

For a linear time-invariant structural system with n degrees of freedom, m-order input, and p-order output, the discrete state-space equations can be represented as follows:

(3) $$\left\{\begin{array}{c} z_{k+1}=Az_{k}+Bu_{k}+w_{k}\\ y_{k}=Cz_{k}+Du_{k}+v_{k} \end{array}\right.$$

where $z_{k+1}={\left[\begin{array}{cc} x_{k+1}^{T} & {\dot{x}}_{k+1}^{T} \end{array}\right]}^{T}$ is the structural state vector at time instant t=(k+1)Δt, $x_{k+1}$ is the displacement vector at time instant t=(k+1)Δt, ${\dot{x}}_{k+1}$ is the n×1 velocity vector at time instant t=(k+1)Δt, A is the n×n system matrix, B is the 2n×m input matrix, $u_{k}$ is the m×1 input vector at time instant t=kΔt, $y_{k}$ is the p×1 output response vector, C is the p×2n output matrix, D is the directed p×m output matrix, and $w_{k}$ and $v_{k}$ are uncorrelated zero-mean stationary white noise sequences with the orders 2n×1 and p×1, respectively, representing process noise (typically caused by interference or modeling inaccuracies) and measurement noise (generally caused by inaccuracies in the sensor measurement data).

The generalized input–output matrix equations were built as follows:

(4) $$Y_{p}=\Gamma_{i}Z_{p}+H_{i}U_{p}+H_{i}^{s}V_{p}$$

(5) $$Y_{f}=\Gamma_{i}Z_{f}+H_{i}U_{f}+H_{i}^{s}V_{f}$$

(6) $$Z_{f}=A^{i}Z_{p}+\Delta_{i}U_{p}+\Delta_{i}^{s}W_{p}$$

where $Y_{p}$ is the past output matrix, $Y_{f}$ is the future output matrix, $U_{p}$ is the past input matrix, $U_{f}$ is the future input matrix, $\Gamma_{i}={\left[C\;CA\;\cdots \;CA^{i-1}\right]}^{T}$ is the generalized observability matrix, $\Delta_{i}=\left[A^{i-1}B\;A^{i-2}B\;\cdots \;B\right]$ is the determined extended controllability matrix, $\Delta_{i}^{s}=\left[A^{i-1}\;A^{i-2}\;\cdots \;I\right]$ is the stochastic extended controllability matrix, $Z_{p}=\left[z_{1}\;z_{2}\;\cdots \;z_{N-2i+1}\right]$ is the historical system state matrix, $Z_{f}=\left[z_{i+1}\;z_{i+2}\;\cdots \;z_{N-i+1}\right]$ is the future system state matrix, p is the subscript, f indicates the future part of the corresponding variable, and the superscript r indicates that the corresponding variable is random.

Through the orthogonal projection of the future output matrix $Y_{f}$ onto the orthogonal complement space $U_{f}^{⊥}$ of the future input data, the generalized observability matrix and the system state vector can be estimated from the orthogonal decomposition result:

(7) $${\hat{\Gamma }}_{i}={\tilde{\omega }}_{1}^{-1}U_{1}S_{1}^{1/2}$$

(8) $${\hat{Z}}_{f}=Z_{f}/U_{f}^{⊥}{\tilde{\omega }}_{2}=S_{1}^{1/2}J_{1}^{T}$$

where ${\tilde{\omega }}_{1}=I$ and ${\tilde{\omega }}_{2}={\left[W_{p}/U_{f}^{⊥}\right]}^{†}W_{p}$.

Subsequently, the system matrix was determined by solving a least-squares problem constructed through the state-space equations, which is expressed as follows:

(9) $$\left[\begin{array}{c} {\hat{Z}}_{i+2}\\ Y_{i+1} \end{array}\right]=\left[\begin{array}{cc} A & B\\ C & D \end{array}\right]\left[\begin{array}{c} {\hat{Z}}_{i+1}\\ U_{i+1} \end{array}\right]+\left[\begin{array}{c} \rho_{w}\\ \rho_{v} \end{array}\right]$$

where $\rho_{w}$ and $\rho_{v}$ are residual matrices.

By employing the least-squares method, one can calculate an estimate for the discrete-time system matrix A, followed by conducting an eigenvalue decomposition on the obtained matrix:

(10) $$A=\Psi \Lambda \Psi^{-1}$$

where $\Lambda =\mathrm{diag}\left[\mu_{r}\right]$ is the diagonal matrix, $\mu_{r}$ is the system eigenvalue, and Ψ is a matrix formed by using the eigenvectors as column vectors.

The r-th order natural frequency ${\tilde{\omega }}_{r}$, damping ratio ${\tilde{\xi }}_{r}$, and mode shape ${\tilde{\phi }}_{r}$ of the structural system can be computed as follows [26,27]:

(11) $${\tilde{\omega }}_{r}=\frac{\sqrt{\left|{\tilde{\mu }}_{r}\right|}}{2\pi },\;{\tilde{\xi }}_{r}=-\frac{{\tilde{\mu }}_{r}+{\bar{\tilde{\mu }}}_{r}}{2\sqrt{{\tilde{\mu }}_{r}{\bar{\tilde{\mu }}}_{r}}},\;{\tilde{\phi }}_{r}=C\Psi_{r}$$

where ${\tilde{\mu }}_{r}$ and ${\bar{\tilde{\mu }}}_{r}$ are complex conjugates, ${\tilde{\omega }}_{r}$ is the natural frequency of the structural system, ${\tilde{\xi }}_{r}$ is the modal damping ratio of the structural system, and ${\tilde{\phi }}_{r}$ is the vibration mode of the structural system.

## 4. Test Result Analysis

### 4.1. Strain Responses and Failure Mode Analysis of the Gravity Dam Model

**Figure 9:**
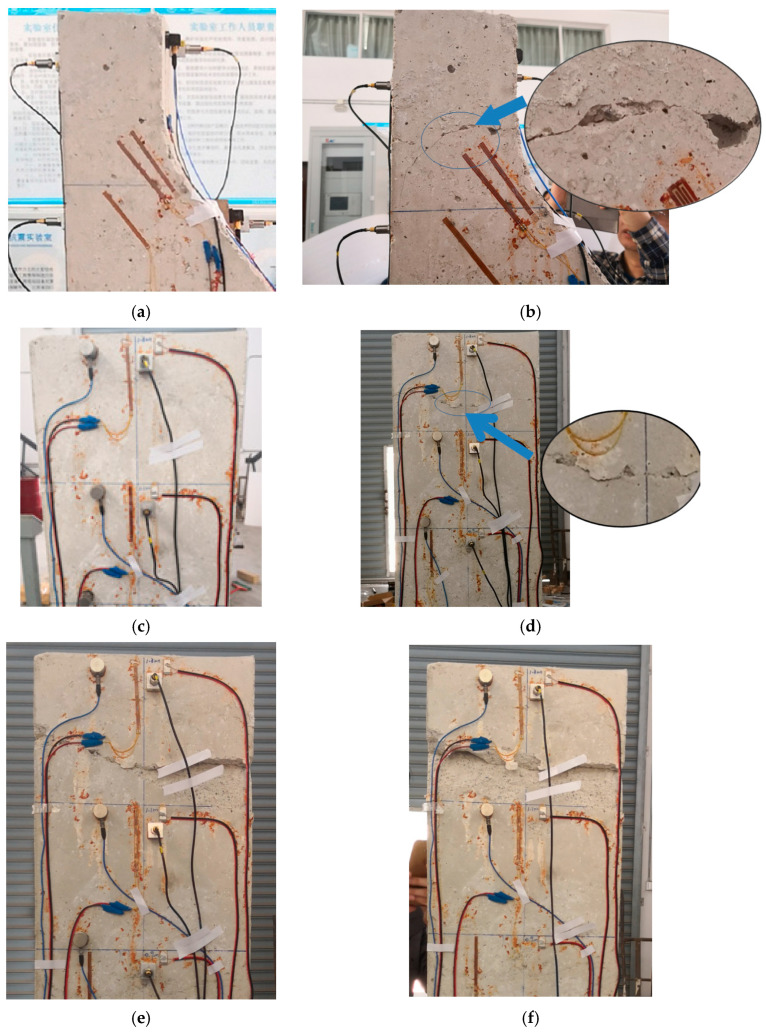
Failure mode of the top portion of the gravity dam model. (**a,c**) Before the experiment. (**b,d**) After condition SE12. (**e**) After condition SE13. (**f**) After condition SE14.

**Figure 10:**
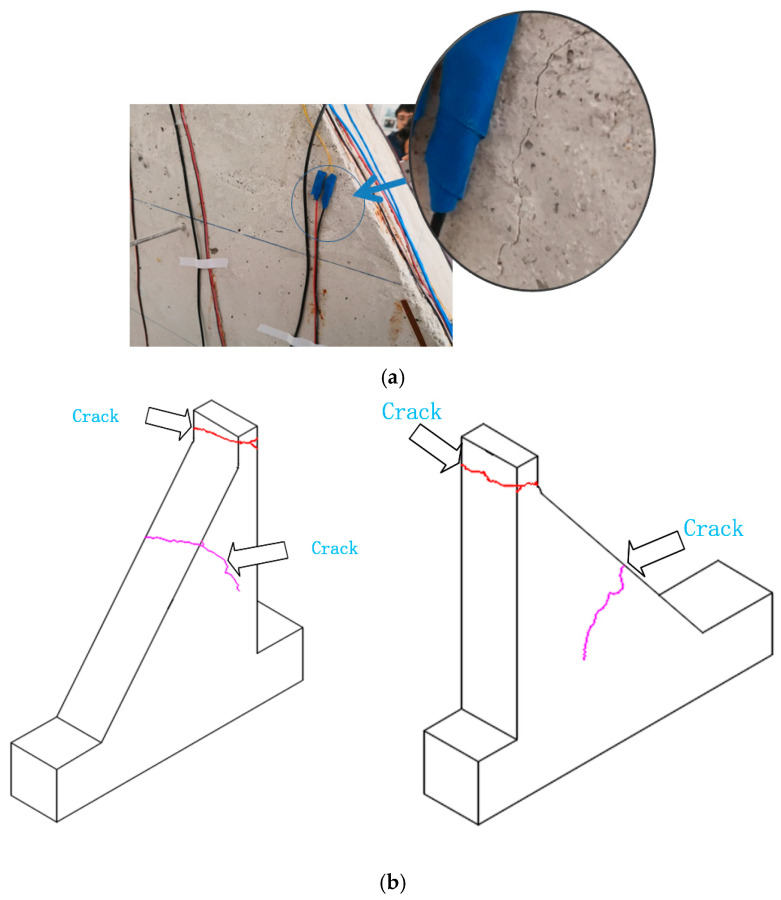
Cracks in the middle of the gravity dam model and the final failure schematic of the gravity dam model. (**a**) Penetration crack in the middle of the gravity dam model (after the SE14 condition). (**b**) Final failure schematic of the gravity dam model.

Under the seismic loads induced by shaking table, damage manifested at both the top and middle portions of the gravity dam model. Figure 9 and Figure 10 show the damage patterns and schematic representations of the ultimate failure at the top and middle of the model, respectively. Based on the damage locations of the gravity dam model, strain response measurement points s1 near the top portion and s5 and s9 near the middle of the model were selected for the analysis. Notably, visible cracks appeared at the top and middle of the gravity dam model under the SE11 and SE13 conditions. Therefore, this study focused on analyzing the SE7, SE8, SE9, SE10, SE11, SE12, SE13, and SE14 conditions.

**Figure 11:**
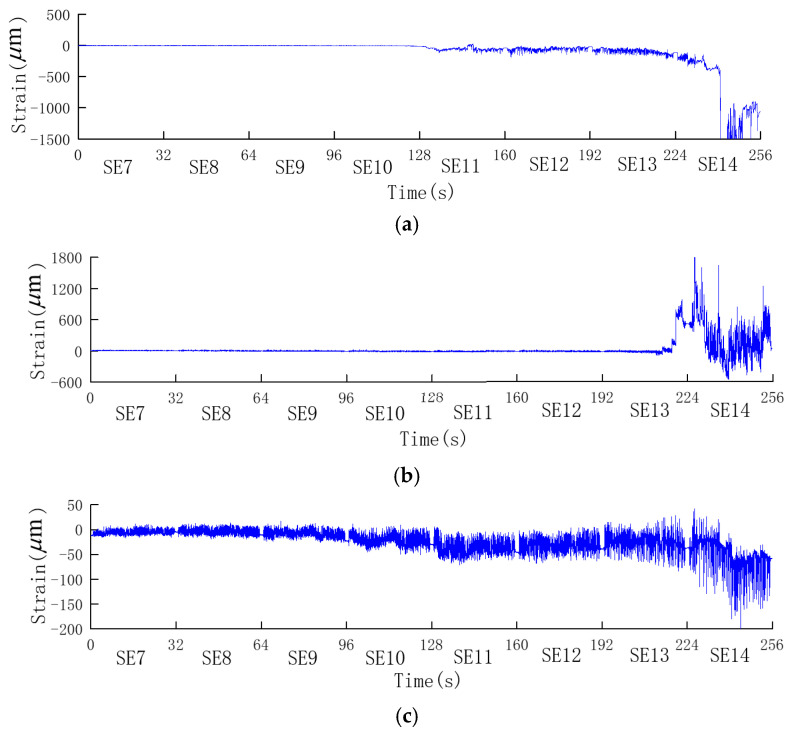
Measured dynamic strain response of the measurement points s1, s5, and s9. (**a**) Measuring point s1. (**b**) Measuring point s5. (**c**) Measuring point s9.

Figure 11 shows the measured dynamic strain responses at measuring points s1, s5, and s9. For the convenience of displaying the actual responses of each point under different conditions, the dynamic strain responses under these conditions were displaced in chronological order. Given that each condition lasted for 32.0 s and the initial time of the SE7 condition was set to 0, the time coordinates for each condition progressively increased. Table 2 lists the strain mean values for the three monitoring points during various time periods under different conditions. During the SE7 to SE10 tests, each monitoring point exhibited small plastic strains with nonzero means, indicating the occurrence of microscopic cracks and damage in the gravity dam model that were imperceptible to the naked eye. Subsequently, under the SE11 to SE14 conditions, the strains at each monitoring point gradually increased.

Specifically, the strain response at the monitoring point s1 experienced various degrees of abrupt changes at multiple time points, especially at *t* = 240.8 s (16.8 s in the SE14 condition), where the strain significantly increased. The measuring point s5 exhibited noticeable abrupt changes starting at *t* = 214.5 s (22.5 s into the SE13 condition), followed by different degrees of changes at subsequent times.

Based on the results of the strain response analysis at each monitoring point, and in conjunction with the damage patterns at the top and middle portions of the gravity dam model shown in Figure 9 and Figure 10, it was evident that microscopic cracks and damage occurred at the top and middle portions of the gravity dam model during the SE7 to SE10 conditions, although these cracks were imperceptible to the naked eye. Under the SE11 condition, visible small cracks appeared at the top of the gravity dam model and continued to expand. After the SE12 condition, the cracks at the top became very pronounced, as shown in Figure 9. In the middle portion of the gravity dam, visible small cracks appeared under the SE13 condition, and after the SE14 condition, further cracks developed in the middle portion, as depicted in Figure 10a.

### 4.2. Analysis of the Acceleration Amplification Coefficients

**Figure 12:**
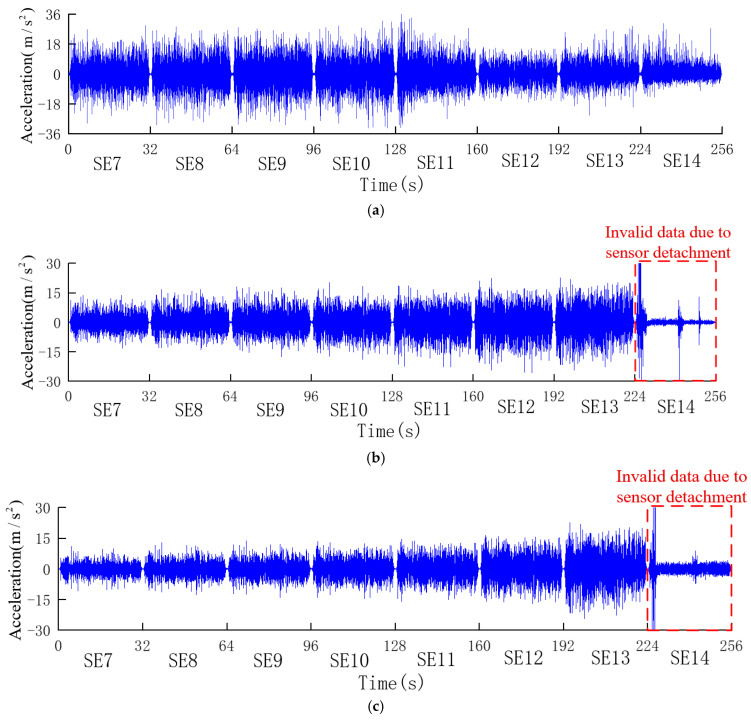

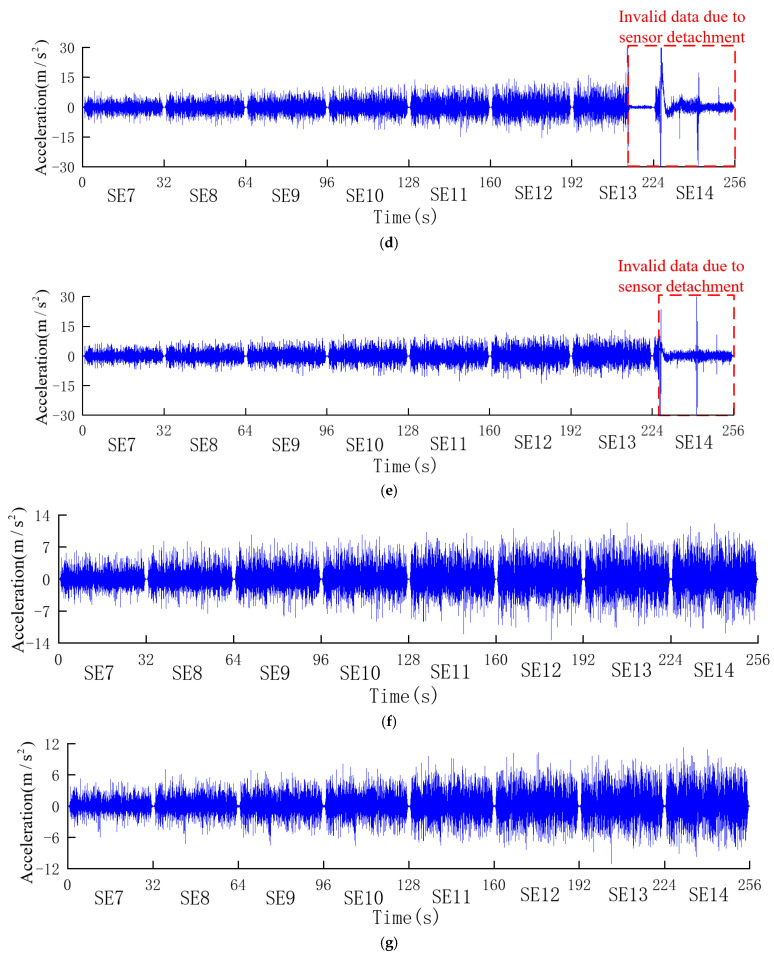
Measured acceleration responses. (**a**) Measuring point a1. (**b**) Measuring point a2. (**c**) Measuring point a3. (**d**) Measuring point a4. (**e**) Measuring point a5. (**f**) Measuring point a6. (**g**) Measuring point a0.

The acceleration responses of the measuring points a1, a2, a3, a4, a5, and a6 on the upstream side of the gravity dam model were selected for analysis, as depicted in Figure 12. Notably, due to the impact of strong seismic loads, the accelerometers a2, a3, a4, and a5 were detached during the SE14, SE14, SE13, and SE14 conditions, respectively. The detached sensors vibrated along the vibration platform, and the data from the corresponding conditions and subsequent measured responses were excluded from the analysis. It is apparent that, with the increase in the elevation, the acceleration responses at these measuring points increase.

The peak acceleration responses (both positive and negative) at the selected accelerometers under different conditions are compiled in Table 3.

**Figure 13:**
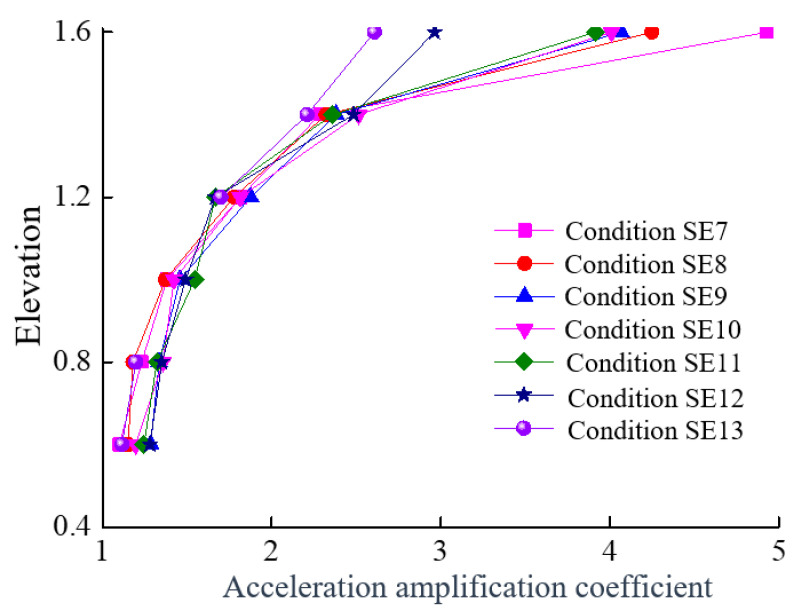
Acceleration amplification coefficients of the selected accelerometers under different conditions.

The absolute values of the peak acceleration responses at each measuring point under different conditions were divided by the absolute value of the peak acceleration of the vibration platform, which resulted in a dimensionless number, representing the acceleration amplification coefficient for each accelerometer under various conditions. Figure 13 shows the acceleration amplification coefficients for the selected accelerometers under different conditions, in which the horizontal axis represents the amplification coefficient and the vertical axis represents the elevation. The elevation of the accelerometers is shown in Figure 3. Figure 8 shows that, with the increase in the elevation, the acceleration amplification coefficient at each monitoring point exhibits an increasing trend. At the dam crest (at the position of accelerometer a1), the amplification coefficient is the highest during the SE7 condition and gradually decreases thereafter. Notably, during the SE12 condition, there is a significant decrease in the amplification coefficient at accelerometer a1, which corresponds to the appearance of visible and substantial cracks at the dam crest during the SE12 condition.

### 4.3. Frequency Spectrum Analysis

**Figure 14:**
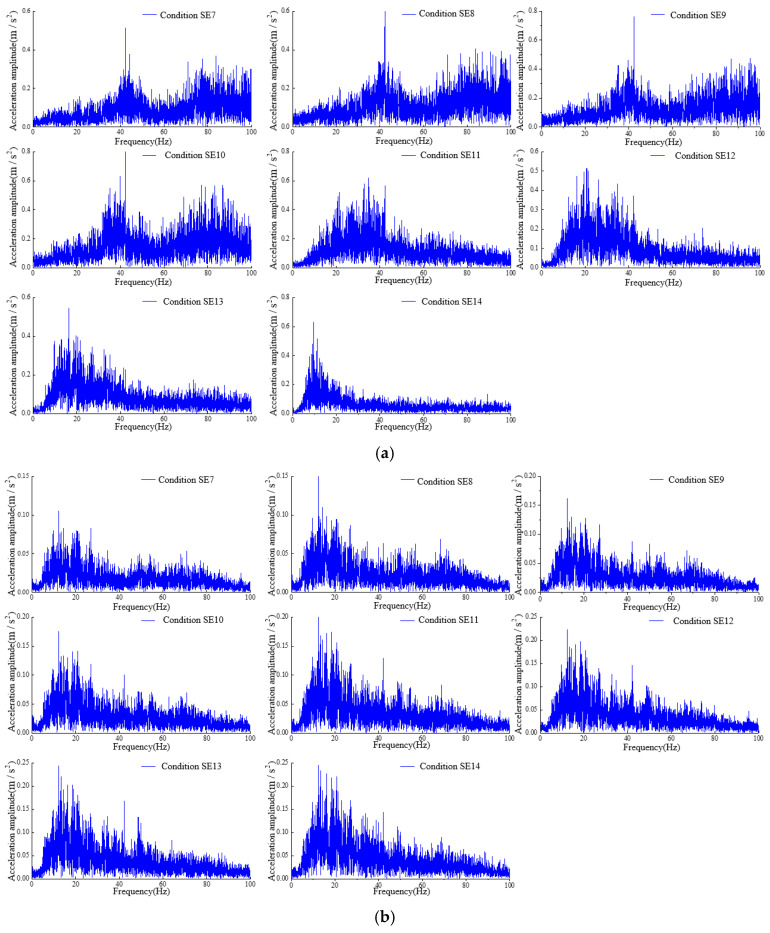
Spectra of the accelerometers a1 and a0 under different conditions. (**a**) Measuring point a1. (**b**) Measuring point a0.

The spectral characteristics of dynamic responses also serve as indicators of structural dynamic properties. Analyzing the variations in these spectral characteristics provides an effective means of assessing structural damage. As previously mentioned, visible cracks appeared at the dam crest in the gravity dam model. The spectra of accelerometer a1 at the dam crest and accelerometer a0 on the surface of the shaking table under different conditions were calculated by utilizing the Fourier-transform method, as depicted in Figure 14. Notably, the peak frequency of the spectrum for point a1 was 42.22 Hz during the SE7 to SE10 transition. Subsequently, during the SE11, SE12, SE13, and SE14 conditions, the peak frequencies were 34.72 Hz, 20.70 Hz, 16.22 Hz, and 9.65 Hz, respectively. This finding is aligned with the observable development of small cracks at the top of the gravity dam model during the SE11 condition and the subsequent conditions.

**Figure 15:**
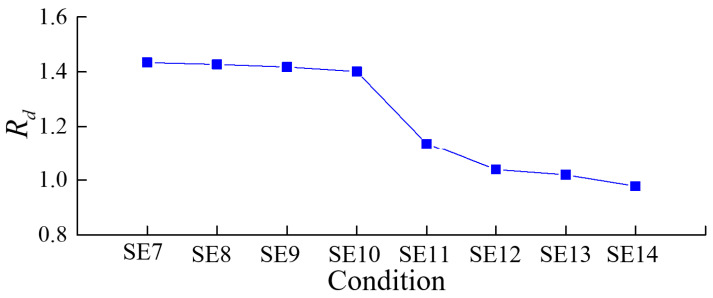
Statistical parameter indicator $R_{d}$ under different conditions.

Then, the statistical parameter indicators $R_{d}$ of accelerometer a1 under various conditions were obtained, as shown in Figure 15. The parameters of acceleration monitoring point a1 gradually decreased as the seismic load progressed. The changes are relatively small during the SE7 to SE10 transition, but a more pronounced reduction is evident in the SE11 extension, followed by a continued decrease. This result is aligned with the fact that the dam exhibits minor damage (microscopic cracks not visible to the naked eye) from SE7 to SE10 and relatively greater damage (cracks visible to the naked eye) from SE11. As structural damage at the dam crest intensifies thereafter, the statistical parameter indicator $R_{d}$ of accelerometer a1 gradually decreases. This also demonstrates the applicability of the statistical parameter indicator $R_{d}$ to identify structural damage in gravity dams. Compared to the acceleration amplification coefficient, the statistical parameter indicator $R_{d}$ is more sensitive to visible crack damage.

### 4.4. Modal Parameter Identification

Based on the measured acceleration responses of the gravity dam model and the accelerometer on the shaking table, numerical algorithms for subspace state space system identification were employed to identify modal parameters for the gravity dam model. In the calculation process, the system order was assumed to range from 2 to 100, with a frequency tolerance of 1%, damping ratio tolerance of 5%, and mode shape tolerance of 2%. The stability criterion was defined by setting the stable point number to 20, signifying that, if more than 20 points exhibited stability, the corresponding mode was deemed stable, and its frequency was regarded as the true frequency. The calculations reveal that the gravity dam model exhibits stable first-order modal parameters only under the SE7 to SE10 conditions. In the SE11 to SE14 conditions, visible cracks at the top of the gravity dam model appeared and gradually developed, leading to the absence of stable modal parameters for the gravity dam. Table 4 presents the first-order frequencies and damping ratios of the gravity dam identified in the SE7 to SE10 conditions. It can be observed that, under the SE7 to SE10 conditions, the first-order natural frequency of the gravity dam model gradually decreases, while the first-order damping ratio gradually increases. This aligns with the pattern of structural damage development, indicating that structural damage results in a decrease in the natural frequency of the gravity dam model and an increase in energy dissipation, reflected in the continuously increasing identified damping ratio.

## 5. Conclusions

This study conducted dynamic failure experimental testing of a gravity dam model on a shaking table and analyzed the structural dynamic characteristics and damage pattern of the dam model. The damage patterns and dynamic characteristics of the gravity dam model were analyzed and compared under different conditions. The main research contents, findings, and future work are as follows:

(1) A model material, named emulation concrete, was selected and prepared; this material consisted of heavy sand, heavy alumina powder, cement, quick-setting agent, and water. This model material exhibited high density, low elastic modulus, and adequate strength, ensuring that the gravity dam model could be smoothly hoisted to the shaking table without damage.
(2) Using the developed emulation concrete material, a gravity dam model was designed and fabricated. Accelerometers and strain gauges were arranged at different locations on the dam body to capture the acceleration and strain responses under various operational conditions. The SE7 to SE14 conditions were selected for the analysis, revealing that the gravity dam model had nonzero mean plastic strains from SE7 to SE10, indicating that microscopic cracks were not visible to the naked eye. Subsequently, visible cracks appeared at the top and middle portions of the gravity dam model. Visible cracks appeared at the top of the gravity dam model during the SE11 condition and continued to develop, and visible cracks in the middle portion of the gravity dam model appeared during the SE13 condition and formed penetrating cracks in the SE14 condition.
(3) The acceleration amplification coefficients of various accelerometers under different conditions were analyzed. The acceleration amplification coefficient of accelerometer a1 at the dam crest noticeably decreased with the progression of seismic loading conditions, indicating a gradually decreasing trend. This aligns with the characteristics of damage appearing near the dam crest and continuously developing. The largest decrease in the amplification coefficient for accelerometer a1 occurred during the SE12 condition, corresponding to visible and significant crack damage near the dam crest. This crack damage affected the transmission of dynamic loads.
(4) The frequency spectrum characteristics of acceleration response point a1 under different conditions were analyzed, and statistical parameters for spectrum characteristics, denoted as $R_{d}$, were proposed for the analysis. The results show that the peak frequency of the spectrum for accelerometer a1 is 42.22 Hz during the SE7 to SE10 conditions, gradually decreasing in the SE11 to SE14 conditions. The parameter $R_{d}$ showed a gradual decreasing trend with the advancement of seismic loading conditions. The parameter $R_{d}$ exhibited a smaller decrease during the SE7 to SE10 conditions and a more pronounced decrease during the SE11 condition, corresponding to the visible small cracks near the dam crest. Clearly, parameter $R_{d}$ is more sensitive to visible small crack damage than is the peak frequency in the spectrum and the acceleration amplification coefficient.
(5) A modal parameter analysis of the gravity dam model was conducted using the N4SID method. Visible small cracks appeared during the SE11 condition, resulting in no stable modes for the gravity dam model under these conditions. However, the gravity dam model exhibited relatively stable modes during the SE7 to SE10 transition. The analysis of the identified values for the first-order frequency and damping ratio during the four conditions revealed that the natural frequency of the gravity dam model gradually decreased with the development of damage, while the damping ratio continuously increased, indicating an increase in energy dissipation due to damage progression.
(6) Future work will consider water loading in the gravity dam model experiment on the newly established underwater shaking table in the Structural Seismic Laboratory at Hohai University, and the damage patterns and dynamic characteristic parameters of the two different experiments will be compared to consider the influence of water loading on the dynamic characteristics and damage patterns of gravity dam structures.

## Figures and Tables

**Table 1:** Loading conditions of the gravity dam model experiment.

Experimental Conditions	Input Waveform	Experimental Conditions	Input Waveform
WN1	White Noise, 0.1 g	SE1	Artificial Seismic Wave, 0.1 g
SE2	Artificial Seismic Wave, 0.2 g	SE3	Artificial Seismic Wave, 0.3 g
SE4	Artificial Seismic Wave, 0.4 g	SE5	Artificial Seismic Wave, 0.45 g
SE6	Artificial Seismic Wave, 0.5 g	SE7	Artificial Seismic Wave, 0.6 g
SE8	Artificial Seismic Wave, 0.7 g	SE9	Artificial Seismic Wave, 0.8 g
SE10	Artificial Seismic Wave, 0.9 g	SE11	Artificial Seismic Wave, 1.0 g
SE12	Artificial Seismic Wave, 1.05 g	WN2	White Noise, 0.1 g
SE13	Artificial Seismic Wave, 1.1 g	SE14	Artificial Seismic Wave,1.2 g
WN3	White Noise, 0.1 g		

**Table 2:** Mean value of the strain response at each measurement point (unit: μm/m).

	Condition	SE7	SE8	SE9	SE10	SE11	SE12	SE13	SE14
Measuring Point									
s1		−3.21	−3.32	−3.73	−5.93	−52.22	−53.56	−86.84	−961.0
s5		5.88	3.09	−2.32	−6.58	−9.83	−12.59	83.56	222.55
s9		−5.94	−4.41	−8.32	−21.82	−36.95	−30.45	−22.66	−41.54

**Table 3:** Peak acceleration at each measurement point under different conditions (unit: m/s^2^).

	Accelerometer	a1	a2	a3	a4	a5	a6
Condition							
SE7	Positive	29.33	13.76	10.94	8.31	7.42	6.62
	Negative	−29.68	−13.14	−10.80	−8.21	−6.62	−6.10
SE8	Positive	28.40	16.74	12.82	9.36	8.51	8.30
	Negative	−30.63	−14.16	−12.68	−9.89	−8.17	−7.52
SE9	Positive	28.07	17.95	13.79	10.35	9.83	9.69
	Negative	−30.66	−17.65	−14.14	−10.99	−10.07	−8.39
SE10	Positive	36.94	20.37	13.46	11.52	11.00	9.69
	Negative	−37.39	−18.91	−14.70	−11.44	−10.10	−9.26
SE11	Positive	37.62	18.71	14.57	13.97	10.98	10.21
	Negative	−32.33	−22.70	−16.04	−14.85	−12.70	−11.97
SE12	Positive	30.62	22.55	16.51	14.31	11.86	11.08
	Negative	−24.40	−25.67	−17.14	−15.41	−13.96	−13.26
SE13	Positive	28.83	22.55	18.16		13.23	12.32
	Negative	−24.69	−24.42	−18.78		−11.46	−10.58
SE14	Positive	30.57					12.13
	Negative	−13.87					−11.75

**Table 4:** First-order frequency and damping ratio of the gravity dam model for conditions SE7 to SE10.

Condition SE7		Condition SE8		Condition SE9		Condition SE10	
Frequency (Hz)	Damping Ratio (%)	Frequency (Hz)	Damping Ratio (%)	Frequency (Hz)	Damping Ratio (%)	Frequency (Hz)	Damping Ratio (%)
42.32	5.67	41.78	6.26	41.47	6.58	40.39	7.72

## Data Availability

Data are contained within the article.
